# Teachers’ Adoption of Emotions-Based Learning Outcomes: Significance of Teachers’ Competence, Creative Performance, and University Performance

**DOI:** 10.3389/fpsyg.2022.812447

**Published:** 2022-06-22

**Authors:** Binbin Cai, Zahid Shafait, Lifeng Chen

**Affiliations:** ^1^International College, Zhejiang University of Technology, Hangzhou, China; ^2^School of Management, Northwestern Polytechnical University, Xi’an, China; ^3^School of Business, Zhejiang University City College, Hangzhou, China; ^4^School of Public Affairs, Zhejiang University, Hangzhou, China

**Keywords:** Pakistan, emotional intelligence, learning outcomes, organizational performance, teacher’s competence, creative performance

## Abstract

Studies have revealed that emotion-based learning outcomes are scarce when teachers’ competence and creative performance are neglected, further university performance in relation to teachers’ emotion-based learning outcomes is disregarded in literature so far. Based on the Attributional theory of achievement motivation and emotion, the purpose of this empirical study is to investigate the effects of Emotional Intelligence (EI) on learning outcomes (social, cognitive, self-growth outcomes, and satisfaction with university experience) of academicians in Pakistan’s higher education institutions (HEIs). This study also examines the mediating role of teacher competence (personal assessment) and creative performance (Creative self-efficacy and leadership/supervisor support) in a relationship between EI and learning outcomes. Furthermore, this study ascertained the relationship between learning outcomes and organizational performance (OP) of HEIs. This study used a sample frame of 237 academic professionals from Pakistani HEIs, the hypothesized associations were ascertained using the partial least squares structural equation modeling method (PLS-SEM). The findings disclose that EI has a positive and significant influence on learning outcomes. Furthermore, an indirect relation between EI and learning outcomes is established through teacher competence and creative performance while the relationship between learning outcomes and OP is established also. Results of the considered study reinforce the academic understanding of EI and propose how academicians of HEIs can value their competence and creative performance which in turn enhances learning outcomes and OP. There is a lack of studies in HEIs that investigate the relationship between EI, teacher competence, creative performance, learning outcomes, and OP. This is one of the initial researches that not only empirically examine the interface of EI, learning outcomes, and OP of HEIs’ academicians but also enlightens comprehensions into the prevailing literature by immediate investigation of the mediating role of teacher competence and creative performance in fundamental association.

## Introduction

Teachers’ professional development has been argued to be crucial for effective teaching and institutional performance (Shafait et al., 2021c). It has been, in particular in Pakistan, established that teachers are owed the chance to improve their skills and knowledge while striving for exploration and application of concurrent professional ideas (Latif et al., 2019). Teachers’ development in Pakistan, furthermore, identifies emotional intelligence (EI) as a vital gauging measure for efficacious learning and performance (Asrar-ul-Haq et al., 2017). However, teachers’ domain in Pakistan regarding their emotional development needs further investigation (Pervaiz et al., 2019; Shafait et al., 2021c).

An attributional theory of achievement motivation and emotion (Weiner, 1985) argued that locus, stability, and controllability with emotional experience motivate/demotivate the personnel in pursuit of learning. The theory, therefore, makes a way for thinking to emulate emotions and actions for learning outcomes (Graham, 1991). Asrar-ul-Haq et al. (2017) and Shafait et al. (2021c) investigated teachers with respect to their EI and performance in HEIs of Pakistan. EI asserts and promotes the teaching process in relation to societal amplification, communication, and better pedagogical alliance; furthermore, teachers’ knowledge and learning are adjudicated heavily through their EI application (Shafait et al., 2021d). Therefore, teachers with EI in HEIs foster learning evolution and strengthen the institute’s overall performance, hence, making certain the availability of the best possible emotionally intelligent crew for HEIs (Woods, 2012). However, EI lacks substantial strategic and literary evidence in HEIs (Shafait et al., 2021d), specifically in Pakistan (Asrar-ul-Haq et al., 2017; Shafait et al., 2021c). Moreover, Zhoc et al. (2018) urged future researchers in higher education to investigate the EI (spread across the operational tiers) in relation to their learning outcomes (social, cognitive, self-growth outcomes, and satisfaction with university experience). Similarly, the emotional realm of teachers involving social, personal, and self-actualizing aspects of being human are still in need of an extended investigation (Dolev and Leshem, 2017), as teachers preach valuable knowledge and skills that make the service experience in HEIs valuable (Latif et al., 2019).

Higher education institutions teach and advocate consistent change and learning (Shafait et al., 2021b). Learning outcomes (social, cognitive, self-growth outcomes, and satisfaction with university experience) of HEIs personnel, therefore, are considered vital in their respective domain (Shafait et al., 2021c). Furthermore, learning outcomes are at the core of HEIs’ accomplishments and their personnel success even away from their institutional life (Zhoc et al., 2018). Moreover, emotions help learning to flourish, convincing the learner to utilize the emotions as motivation rather than an unfavorable signal, and hence fostering the desired learning outcomes (Shafait et al., 2021d). Similarly, teachers’ EI certainly assists them to maximize their learning outcomes, numerous studies likewise, comprehended that EI heavily contributes to enhanced learning outcomes (Zhoc et al., 2018; Shafait et al., 2021c).

Latif et al. (2019), recognized in the Pakistani context that teachers need to continuously improve their professional skills, expand their knowledge and remain upright with new professional ideas. McCroskey (1992), explained the three vital dimensions of a credible teacher as competence, trustworthiness, and being seen as caring. We, though, here examine only the first dimension. Teacher competence retains knowledge or expertise in a specific area, as well as encompassing complex material explanation, better class management skills, abrupt response to students’ queries, and efficient communication (Biesta, 2017; Willis and Klenowski, 2018), hence, standardizing certain measures for the teacher personal assessment and learning in HEIs (Sadler and Reimann, 2018). If a teacher is balanced in grading, approves and maintains fair treatment with instant feedback, and refrains from any sort of embarrassment of or by students unless they are seen as less competent (Miller et al., 2017), the personal assessment of teachers being competent incumbents is at stake (Box et al., 2015). Teachers are evaluated depending on their competence in relation to their gains in terms of emotional and cognitive composure (Mérida-López and Extremera, 2017). Based on the emerging links between EI and teaching, it is suggested that teachers who desire to become more effective and competent can benefit from professional opportunities to develop their EI (Dolev and Leshem, 2017). Furthermore, teachers’ ability to manage their emotional and cognitive challenges can make them the best fit, more effective, and competent in any given circumstances (Shafait et al., 2021c). Zhoc et al. (2018), argued that positive emotion endurance improves problem-solving skills, and facilitates recall of affectively neutral and positive information with improving decision-making skills and overall learning outcomes. Furthermore, it is argued to get teacher’s competence as a catalyst to formulate a model of learning in HEIs with the introduction of EI of stakeholders on one side (Leighton et al., 2018). Leighton and Bustos Gómez (2018), asked future researchers to apply and investigate teachers’ competence; therefore, this recommendation is carried forward with academicians of HEIs of Pakistan.

Dynamic professional assignments oblige personnel to manage constant creative solutions while fostering learning and improving performance; HEIs therefore are educating and promoting creative performance in their instant stakeholders (Shafait et al., 2021c). Creative performance, thus, is crucial for HEIs’ sustainable competitive performance (Iqbal et al., 2019). Creative self-efficacy and leadership/supervisor support are two vital components of creative performance (Shalley and Gilson, 2004; Walumbwa et al., 2018) that are taken as indicators of creative performance in this study. A recent study investigated the association between personnel EI and their creativity, establishing that EI is vital for creative performance (Darvishmotevali et al., 2018) exclusively in the education sector (Siu and Wong, 2016). The education sector, however, lacks a realistic literary indication regarding EI and creative performance (Rodrigues et al., 2019). Likewise, Rubenstein et al. (2018), argued that future researchers to cater creative performance in HEIs practically. Similarly, Greene et al. (2019), enticed the practitioners in the field to investigate the creative performance and learning outcomes in HEIs.

Latif et al. (2019), substantiated that teachers are the primary source of university performance and competitive advantage. Furthermore, teachers with enhanced learning outcomes contribute heavily to the research and development of the university (Sahibzada et al., 2019), therefore, there should be personnel regular performance and learning checks, they continued, to garner their learning and contribution toward university performance. However, teachers are ignored in relation to their systematic internal management, learning and outcomes in HEIs of Pakistan (Latif et al., 2019), which deteriorate university performance (Iqbal et al., 2019). HEIs, therefore, are meant to facilitate the learning growth and outcomes of teachers for better institutional performance (Sahibzada et al., 2019). Park and Jacobs (2011) urged future researchers to investigate organizational performance in relation to personal learning outcomes, furthermore, Iqbal et al. (2019) and Sahibzada et al. (2019), suggested undertaking the university performance as an endogenous variable. This study, to be precise, attempts to discourse the underneath research endeavors:

*RQ1:* Is there a direct impact of EI on the learning outcomes of academicians in HEIs of Pakistan?

*RQ2:* Do teacher competence and creative performance mediate between EI and learning outcomes of academicians in HEIs of Pakistan?

*RQ3:* Is there a direct impact of academicians’ learning outcomes on OP in HEIs of Pakistan?

## Literature Review

### Why Is Higher Education Still Confused About Teacher Emotional Intelligence?

Higher education institutions are constrained by a specific paradigm, based on the limited acknowledgment of teachers’ abilities, which hinder unearthing the individuals’ multiple intelligences including EI (Sartori et al., 2015). Furthermore, Teachers’ EI is vital for effective teaching, however, educational institutes are still lacking the proper strategies to prevent burnout in teachers (Asrar-ul-Haq et al., 2017). Burnout has three indications and one of them is Emotional Exhaustion (EE), conceived as the feeling of being physically and emotionally overextended, hence, there is no clear method to overcome these flaws in teachers (Mérida-López and Extremera, 2017). Moreover, Shafait et al. (2021c) argued there is a scarcity of research regarding teaching in higher education and the emotional experiences of lecturers, even though it is an important factor. In the United Kingdom, Mortiboys (2013) is one of the few authors who directly highlighted the importance of EI for teaching in higher education relating to learning outcomes and behavior management. Shafait et al. (2021d) argued that HEIs’ teachers can develop greater sensitivity, heightened solidarity, and stronger reflexivity through EI. Zhoc et al. (2018), explained that EI is a reciprocities phenomenon that guarantees the improved collegial relationship in HEIs.

Higher education institutions’ face numerous constant challenges, especially in Pakistan, despite the fact that technological and educational reforms are on the upper verge (Asrar-ul-Haq et al., 2017). Some vital challenges, they continued, are the declining quality of education, the non-serious attitude of the HEIs customers, and the heavy workload on staff, which in turn make it difficult for the HEIs’ professionals, especially teachers, to come along with the academics as well as societal demands of customers i.e., parents and students. Ignat and Clipa (2012), argued that if HEIs’ servers are determinant enough to develop their professional and emotional competencies, then such challenges can be met easily.

### Can Emotional Intelligence Be Set as a Norm in Higher Education?

Higher education institutions are meant to be the industries of transformation in relation to learning and intellect (Latif et al., 2019). HEIs, therefore, should consider their clients while incorporating learning designs that can foster their personal, emotional, academic, social, and professional progress (Iqbal et al., 2019). HEIs nowadays are encouraging learning outcomes tangled with EI with a vital focus on the social process of enrichment, communication, and collaboration with quality and continuous improvement (Zhoc et al., 2018). Therefore, it is imperative to argue that HEIs commodification is largely mediated by emotions (Shafait et al., 2021c). If a constructive environment is open for the university personnel for their enhanced emotional management, the proficiencies of diverse assignments, hence are reinforced comprehensively for operational and relational assignments (Woods, 2012). Hence, central learning competencies encourage the various intelligences and explicit contemporary talents that are available in the university interceptions (Zhoc et al., 2018). Gilar-Corbi et al. (2018), proposed the “Emotional Intelligence Training Program” (EITP) for HEIs to nurture solutions to the possible gaps in the course of excellence.

Higher education institutions, around the globe, are encouraging a paradigm based on different learning models for assimilation of diverse competencies including EI (Gilar-Corbi et al., 2018; Shafait et al., 2021a). This approach enables the HEIs stakeholders to mature their capacity for progress, elasticity, and surviving hardships while nurturing their professional and personal betterment (Bowen, 2018). Moreover, EI can be enforced in HEIs through interrelation between employees and the organization in terms of knowledge and learning (Shafait et al., 2021d), having interactive relations with employees after their emotional assessment (Asrar-ul-Haq et al., 2017), having adequate information about the employees’ mindful care for institutions and institution’s reciprocal effective service initiatives engraved with EI (Akram et al., 2017), arranging EI training sessions with an intent to foster knowledge networking (Dolev and Leshem, 2017), utilizing EI as an enabler for incumbents’ motivation with approachable internal information frameworks and a fair interplay of ideas, information and EI, looking at knowledge management through EI as a competitive advantage in HEIs, and recognition and rewards to promote innovation and knowledge management processes (Shafait et al., 2021c).

### Hypotheses Development and Conceptual Framework

This study is inferred on the basis of the attributional theory of achievement motivation and emotion (Weiner, 1985). This theory substantiates the casual insights of locus, stability, and controllability for learning while comprehending the intentionality of personnel in the process. Emotional experience, therefore, is influenced by the causality effects of mentioned three factors, hence motivating/demotivating the personnel for the learning and success. This theory, therefore, unites the emotions and actions in order to align the motivations of personnel for the optimized learning outcomes. Based on the ascribed theory’s explanation, the considered study strives to investigate the thoroughly researched framework of teachers’ EI, learning outcomes, competence, creative performance, and OP.

### Emotional Intelligence, Teacher Competence, and Learning Outcomes

Teachers’ professional expansion is seen as indispensable for their personal and institutional competence and well-being (Latif et al., 2019). Teacher competence is all about the awareness of content and pedagogic knowledge, instructional and class management capabilities, reflection, communication, care and motivation, encouraging learning settings, and stimulating students’ eagerness (Dolev and Leshem, 2017). EI, therefore, is at the base of teachers’ professional development, as teachers sometimes experience emotions during their professional assignments (Pervaiz et al., 2019) which impact their mentality and attitudes toward students, respectively (King and Chen, 2019). Hence, it is imperative for teachers to be competent and to incur and develop EI from available institutional opportunities (Shafait et al., 2021c). Furthermore, EI is vital for teacher competence to counter uncertain institutional policy shifts and to tackle the colleagues/customers with depression or negative behaviors (Goleman, 1995). Day et al. (2007), endorsed Goleman’s research explaining EI as vital during the course of a teacher’s effective competence incorporation. Teachers’ competence, moreover, allows them to develop supporting relations with students, allowing them to strengthen their abilities with intrinsic motivation, work collaboratively and fostering learning outcomes (Jennings and Greenberg, 2009).

Leighton and Bustos Gómez (2018), encouraged future studies to pursue teacher competence as a mediator, while a recent study did use teacher competence as a mediator (Sutherland et al., 2018). Since it was conducted in a school setting, therefore, this study is applying it to Pakistan’s higher education. Therefore, we can deduce the hypothesis based on the provided arguments;

**H1a:** There is a significant influence of Emotional Intelligence on teachers’ competence.

**H1b:** There is a significant influence of teachers’ competence on learning outcomes (cognitive, social, self-growth outcomes, satisfaction with university experience).

**H1c:** Teachers’ competence mediates between emotional intelligence and learning outcomes (cognitive, social, self-growth outcomes, satisfaction with university experience) of academicians in Pakistan HEIs.

### Emotional Intelligence, Creative Performance, and Learning Outcomes

Emotional intelligence and creativity are interdependent, EI therefore, induces professionals to correctly handle their assignments with new and useful ideas (Darvishmotevali et al., 2018). Furthermore, EI balances the relation between mood and performance, hence letting creative performance come into play (Jafri et al., 2016). The education sector, similarly, promotes the interrelation of teachers’ EI and their creative performance (Shafait et al., 2021c). Shafait et al. (2021d) established that emotionally intelligent HEIs professionals channel either positive or negative emotions to resolve the fatigues on hand, while Othman and Tengku Muda (2018) endorsed the very idea of Shafait. It is further established the relationship between EI and creative performance argues that personnel with a higher level of EI produce higher standards of creative performance (Darvishmotevali et al., 2018). However, Ramy et al. (2014) do not support the direct relationship between EI and creativity.

**Table:** 

Teacher competence in relation with their EI and learning outcomes.			

	Subdimensions of EI	Literary evidence regarding teachers’ EI	Literary evidence regarding teachers’ learning outcomes

Emotional intelligence	Awareness evaluation and expression of emotions	Consideration of emotional information (Dolev and Leshem, 2017)	Kyndt et al., 2016
		Emotional comprehension (Asrar-ul-Haq et al., 2017)	Thurlings and den Brok, 2017
	Emotional simplification of rational	Dissimilar situations ask to apply different emotions (Pervaiz et al., 2019)	García, 2016
		Emotions support learning (King and Chen, 2019)	
	Thoughtful and analytical of emotional evidence	Teaching manifests the range of emotions due to the volatile scenarios on hand (Mérida-López and Extremera, 2017)	de Ruiter et al., 2019
	Emotions’ regulation	Positive emotional display of teachers with their self-control, job satisfaction and more support/power from colleagues (Asrar-ul-Haq et al., 2017).	Pogere et al., 2019

Learning outcomes, for the considered study, are social outcomes (communication skills, leadership, and teamwork) cognitive outcomes (critical and analytical thinking, problem-solving) self-growth outcomes (time management and critical self-reflection), and satisfaction with university experience (Zhoc et al., 2018). In the 21st century, additionally, HEIs are promoting creativity, critical and rational thinking, problem-solving, and decision making as vital learning outcomes (UNESCO, 2016). Fairweather and Cramond (2010), further investigated and established that knowledge and problem-solving efforts foster the creative performance of HEIs’ personnel. Similarly, studies in HEIs validated a positive relationship between the stated learning outcomes and the creative performance of incumbents (Nami et al., 2014; Rogaten and Moneta, 2016). Therefore, we can deduce the hypothesis based on the provided arguments;

**H2a:** There is a significant influence of EI on the creative performance of academicians of Pakistan HEIs.

**H2b:** There is a direct significant influence of creative performance on learning outcomes (cognitive, social, self-growth outcomes, and satisfaction with university experience) of academicians of Pakistan HEIs.

**H2c:** Creative performance mediates between EI and learning outcomes (cognitive, social, self-growth outcomes, and satisfaction with university experience) of academicians in Pakistan HEIs.

### Learning Outcomes and Organizational Performance

Delery and Doty (1996) investigated three perspectives while explaining the relationship between learning outcomes and organizational performance: (1) universalistic i.e., KSAs i.e., knowledge, skills, and abilities; (2) contingency i.e., organizational policy/strategy, and (3) configurational i.e., motivation, commitment, etc. Organizational performance, therefore, is dependent on personnel learning outcomes (Park and Jacobs, 2011; Yasmin et al., 2019). Numerous studies, whether educational or commercial, have shown that various outcomes result from workplace learning including knowledge, skills and abilities, motivation, organizational commitment, job performance, organizational performance, transfer of learning, and motivation to transfer learning, personal growth, and sociability (Zhoc et al., 2018; Pervaiz et al., 2019). In HEIs, it is seen as necessary for personnel to enhance their learning and maintain the pace with concurrent social demands, likewise, organizational attractiveness and performance are imperative to comprehend the set goals. Educational institutions and personnel, therefore, can be assisted through supplementary informational, professional, and judgmental resources (Sahibzada et al., 2019) for enhanced individual learning and institutional performance (Iqbal et al., 2019). Therefore, the learning capability of educational professionals can be improvised, hence improved through the provision of opportunities for their personal learning and betterment of overall organizational performance (Sergiovanni, 1995). Therefore, we can deduce the hypothesis based on the provided arguments;

**H3:** There is a significant influence of learning outcomes on the OP of academicians of Pakistan HEIs.

Moreover, the conceptual framework is mentioned in the consequent part of this paper before the conclusion section.

## Methodology

### Research Universities

Knowledge-centered economies devote extra attention to HEIs development through research and creativity directed social and economic development (Veer Ramjeawon and Rowley, 2017). Pakistan thus needs its HEIs to excel in research and innovation (Iqbal et al., 2019). The Higher Education Commission of Pakistan, therefore, is extending its practical measures to encourage research culture in HEIs (Shafait et al., 2021c). However, these measures are still insufficient for a better and more dynamic research culture, therefore, teachers’ EI, learning, and HEIs’ performance are at the core of a counter strategic plan to undo the educational challenges (Asrar-ul-Haq et al., 2017; Iqbal et al., 2019). Furthermore, the considered study is meant to execute the relationship between EI, learning outcomes, teacher competence, creative performance, and HEIs’ performance in Pakistan.

### Population, Sample and Data Collection

Teachers from HEIs of Pakistan (Islamabad and Peshawar) were taken as the target population for this study. The survey questionnaire was applied to collect the data in order to examine the logical hypothesized relationships. The convenience sampling technique is used for the data collection. The said technique is an inexpensive route for data collection, furthermore, utilized in business and social research comprehensively (Iqbal et al., 2019; Shafait et al., 2021c). Initially, 500 questionnaires were circulated, however only 263 questionnaires were returned, giving a 52.6% response rate. Further, 26 invalid questionnaires were discarded, hence, the remaining 237 surveys were taken into consideration for the final statistical assessment. Furthermore, the time period for data collection spread from April 2019 – June 2019. The remaining sample size is fair enough to determine the complex path model, as the case with opted research model, through Structural Equation Model (SEM; Shafait et al., 2021c). Further, the explanation of demographic variables is represented in Table 1.

**TABLE 1:** Demographic variables.

Demographic variables	Frequency	Percentage
Questionnaires sent/delivered	500	100
Questionnaires received back	263	52.6
Discarded	26	5.2
Questionnaires useful for analysis	237	47.4
**Province**		
Islamabad	107	45.1
Peshawar	130	54.9
**Gender**		
Male	134	56.5
Female	103	43.5
**University Name**		
Abasyn University	16	6.7
City University	13	5.5
Institute of Management Sciences	16	6.7
Islamia College University	15	6.3
University of Engineering and Technology	12	5.1
Shaheed Benazir Bhutto Women University	14	5.9
CECOS University	17	7.2
Qurtaba University	13	5.9
Sarhad University	15	6.3
Bahria University	17	7.2
Comsats University	13	5.9
Institute of Space Technology	17	7.2
Riphah University	15	6.3
Air University	16	6.7
International Islamic University	13	5.9
Shaheed Zulfikar Ali Bhutto Institute of Science and Technology	15	6.3

### Measures of the Concepts

This research study employed 66 measurement items that were adopted from the literature. Minimal phrasal modifications, however, captured to envision the university context (Shafait et al., 2021d). Additionally, the questionnaire utilized a five-point Likert scale stretching from “1” meaning “strongly disagree” to “5” meaning “strongly agree.” Table 2, furthermore, reflects the sources of engaged measurement instruments.

**TABLE 2:** Source of measurement instruments.

Variable	Dimensions	No. of items	Source
Emotional intelligence		33	Schutte et al., 1998
Teacher competence		6	Teven and McCroskey, 1997
Creative performance		6	Wang and Netemeyer, 2004
Learning outcomes	Cognitive outcomes Social outcomes Self-growth outcomes Student satisfaction with university experience	5 5 5 1	Zhoc et al., 2018 Zhoc et al., 2018 Zhoc et al., 2018 Zhoc et al., 2018
University performance		5	Wang and Wang, 2012

### Data Analysis Procedure

The considered study utilized the quantitative technique with a cross-sectional research design. The partial least squares structural equation modeling (PLS-SEM) technique was employed to analyze the data through the SmartPLS 3.2.7 software package (Ringle et al., 2005). PLS-SEM is trending in business, management, and social sciences research for data analysis, furthermore, it is seen as reliable data analysis tool to analyze the small sample size and non-normal data (Hair et al., 2016). Moreover, PLS-SEM primarily intends to analyze the prevailing theories with multi-layer structural models (Ringle et al., 2018). Principally, PLS-SEM undergoes two phases of statistical analysis i.e., measurement model specification and structural model evaluation (Iqbal et al., 2019). Measurement model specification makes sure a smooth drive for structural model only after a precise analysis of the constructs and carries forward the constructs with good indicator loading, convergent validity, composite reliability (CR), and discriminant validity. Structural model evaluation, on the other hand, is responsible to evaluate the path coefficients with an analysis of their significance utilizing bootstrapping technique.

#### Measurement Model Assessment

In order to endorse the reliability and validity of the constructs and their dimensions, the measurement model was readied in line with the submissions of Hair (2006). The included pool of 69 indicators was adjudicated unharmed/intact from elimination as factor loadings were above or close to the approved limit of 0.60. Henceforth, paving a way for 66 items to be included in the final measurement model. Table 3 shows respective factor loadings for 66 included indicators. Likewise, the values of AVE and CR of opted constructs are equivalent to or exceeding the approved values of 0.50 and 0.70, respectively, hence, establishing the convergent validity and reliability. Discriminant validity, furthermore, is ascertained according to the customary criterion of Fornell and Larcker (1981), which is shown through Table 4. It is, henceforward, determined through confirmatory factor analysis that measurement model assessment is satisfactory for the assessment of the structural model.

**TABLE 3:** Item loadings, reliability, and convergent validity.

	Λ	α	CR	AVE
Emotional intelligence		0.971	0.973	0.527
EI1	0.668			
EI2	0.704			
EI3	0.722			
EI4	0.768			
EI5	0.703			
EI6	0.636			
EI7	0.767			
EI8	0.789			
EI9	0.723			
EI10	0.790			
EI11	0.696			
EI12	0.732			
EI13	0.761			
EI14	0.557			
EI15	0.818			
EI16	0.809			
EI17	0.745			
EI18	0.692			
EI19	0.731			
EI20	0.831			
EI21	0.734			
EI22	0.746			
EI23	0.686			
EI24	0.560			
EI25	0.683			
EI26	0.666			
EI27	0.762			
EI28	0.794			
EI29	0.665			
EI30	0.736			
EI31	0.735			
EI32	0.811			
EI33	0.635			
**Learning outcomes**				
*Cognitive outcomes*		0.843	0.889	0.617
CO1	0.694			
CO2	0.832			
CO3	0.805			
CO4	0.828			
CO5	0.759			
*Social outcomes*		0.854	0.896	0.633
SO1	0.796			
SO2	0.843			
SO3	0.785			
SO4	0.793			
SO5	0.756			
*Self-growth outcomes*		0.792	0.857	0.547
SGO1	0.730			
SGO2	0.789			
SGO3	0.749			
SGO4	0.649			
SGO5	0.774			
*Satisfaction with university experience*		1.000	1.000	1.000
SUE1	1.000			
*Teacher competence*		0.809	0.863	0.512
TC1	0.692			
TC2	0.772			
TC3	0.739			
TC4	0.655			
TC5	0.720			
TC6	0.709			
*Creative performance*		0.818	0.869	0.527
CP1	0.679			
CP2	0.785			
CP3	0.641			
CP4	0.678			
CP5	0.790			
CP6	0.770			
Organizational/university performance		0.765	0.844	0.529
UP1	0.803			
UP2	0.812			
UP3	0.769			
UP4	0.740			
UP5	0.446			

**TABLE 4:** Discriminant validity (Fornell and Larcker criterion).

	CO	CP	EI	OP	SGO	SO	SUE	TC
CO	**0.785**							
CP	0.724	**0.726**						
EI	0.728	0.701	**0.726**					
OP	0.651	0.724	0.705	**0.727**				
SGO	0.727	0.665	0.713	0.650	**0.740**			
SO	0.719	0.708	0.723	0.636	0.734	**0.795**		
SUE	0.561	0.550	0.672	0.513	0.686	0.594	**1.000**	
TC	0.783	0.711	0.714	0.669	0.702	0.714	0.632	**0.715**

*The data in the diagonal (in italic) is the square root of AVE of the construct.*

#### Structural Model Assessment

The structural model assessment was executed right after the assessment of the measurement model. The very assessment is performed for hypotheses analysis through the successive steps. EI’s direct effects, firstly, on the learning outcomes were scrutinized. Thereafter, EI’s direct effects, secondly, on teacher competence and creative performance, furthermore, the effects of teacher competence and creative performance were tested in relation to the learning outcomes of academicians were investigated. Subsequently, the direct effects of learning outcomes on university performance were observed. Furthermore, the significance of direct path and standard error estimation was ascertained using the Bootstrap resampling method with 5,000 resamples (Ringle et al., 2005), Table 5, therefore, shows the results for analyzed direct associations. Consequently, teacher competence and creative performance as mediators were analyzed to weigh the effects of EI on learning outcomes.

**TABLE 5:** Results of structural model path coefficient (direct relationships).

Hypotheses	Relationship	*B*	SD	*t*-value	*P*-values	Decision
H1	EI → LO	0.507	0.055	9.349	**0.000**	Supported
H2	EI → TC	0.802	0.064	12.727	**0.000**	Supported
H3	TC → LO	0.384	0.060	6.363	**0.000**	Supported
H4	EI → CP	0.791	0.065	12.375	**0.000**	Supported
H5	CP → LO	0.092	0.073	1.219	**0.223**	Rejected
H6	LO → UP	0.692	0.101	7.044	**0.000**	Supported

*EI, emotional intelligence; LO, learning outcomes; TC, teacher competence; CP, creative performance; UP, university performance. The meanings of bold values in asked tables are provided this time around in the respective sections while elaborating the tables’ extracted figures.*

#### Mediation Analysis

The hypothesized mediation, finally, of teacher competence and creative performance between EI and learning outcomes were analyzed through the Preacher and Hayes (2008) method that is appropriate when applied with the PLS-SEM bootstrapping method (Hair et al., 2016). Memon et al. (2018) urged the researchers to follow Preacher and Hayes’s (2004; 2008) approach and bootstrap the sampling distribution of the indirect effect. Specifically, bias-corrected bootstrapping is considered a powerful method to detect mediation. A statistically significant indirect effect (*t*-value > 1.96, two-tailed, *p* < 0.05) should be taken as an evidence for mediation (Preacher and Hayes, 2004; Zhao et al., 2010).

Nevertheless, recent development in mediation literature unequivocally discourages researchers from using Baron-Kenny’s approach because of its severe limitations (Aguinis et al., 2016; Green et al., 2016; Memon et al., 2018). These limitations include: (1) low statistical power, (2) not directly testing the significance of a specific indirect effect, (3) neither quantifying the magnitude of the mediation effect, nor accommodating models with inconsistent mediation (MacKinnon, 2000; MacKinnon et al., 2002; Hayes, 2009; Rungtusanatham et al., 2014). Considering these limitations, ‘using Baron and Kenny’s approach might produce misleading results, refute potentially significant theoretical relationships, and in turn damage future theory building’ (Rungtusanatham et al., 2014, p. 131).

Despite the mediation insertion, the direct effects of EI and learning outcomes were found significant and positive (β = 0.51, *p* < 0.001). Indirect effects of teacher competence and creative performance were analyzed as significant (β = 0.309, *p* < 0.001; β = 0.072, *p* < 0.001), respectively. Thus, comprehending the full and partial mediation for the considered variables. The results substantiated and show that the effect of EI on learning outcomes passes partially through teacher competence and creative performance. Results of the mediation analysis are presented in Table 6.

**TABLE 6:** Summary of mediation results.

	Indirect path						
Hypothesis	Path	β	Path	β	Mediation effect β	*t*-value	Decision
*H7a*	EI → TC	0.802	TC → LO	0.384	0.309	1.217*	Supported
*H7b*	EI → CP	0.791	CP → LO	0.092	0.072	5.280	Supported

Bootstrapping (n = 500). *P < 0.001.

*The meanings of bold values in asked tables are provided this time around in the respective sections while elaborating the tables’ extracted figures.*

**FIGURE 1:**
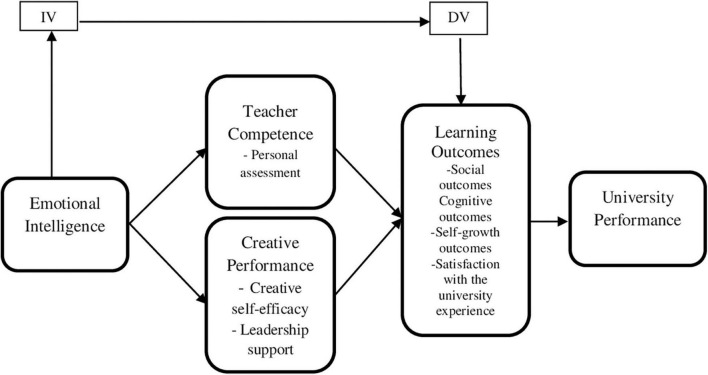
Conceptual framework.

## Discussion, Conclusion, and Practical Implications

### Discussion

The considered research investigated the effect of EI on learning outcomes (social, cognitive, self-growth outcomes, and satisfaction with university experience) of teachers in Pakistan HEIs, the indirect effect of teacher competence and creative performance between EI and learning outcomes while a direct effect of learning outcomes on organizational/university performance. The findings of this study subsidize the literature in multiple ways. First, this study approves the certainty of EI to facilitate the learning outcomes of teachers in HEIs. Findings of considered research establish that EI significantly and positively affects learning outcomes (social, cognitive, self-growth outcomes, and satisfaction with university experience). Furthermore, these results endorsed the previous findings by Zhoc et al. (2018), however, those were conducted with a focal focus on the HEIs’ students. HEIs, in the modern world, create value through available knowledge and skills to remain competitive in the circle (Shujahat et al., 2019). Moreover, firms are eager nowadays to hire personnel with a fair degree of EI to cope with pressure-paced chaos and to develop the mechanisms for learning outcomes (Zhoc et al., 2018).

Furthermore, this study endorsed the assumed hypothesis that teachers’ competence is well connected with EI and the learning outcomes of teachers in HEIs. The findings validated the previous research results (Corcoran and Tormey, 2012; Dolev and Leshem, 2017). Moreover, teachers take advantage of available professional prospects to develop their EI (Dolev and Leshem, 2017), hence, maximizing their learning outcomes and fostering institutional performance (Thurlings and den Brok, 2017; Iqbal et al., 2019). Additionally, teachers’ competence as mediators (Sutherland et al., 2018) argues the possessors are the obligor of persistent EI development and learning improvements (Jennings and Greenberg, 2009; García, 2016).

Similarly, the study substantially validated the findings regarding the teachers’ EI and their creative performance, hence endorsing the study from Siu and Wong (2016), however, the results failed to validate the relationship between teachers’ creative performance and their learning outcomes in HEIs. Hence, this study is a contradictory piece of research in relation to Li et al. (2017). The subjugation of inadequate opportunities in practive and the discouragement for creative personnel performance in HEIs is alarming (Alencar et al., 2017), which, argues for the proper addressing of creativity and training of EI for HEIs’ incumbents (Rodrigues et al., 2019), hence, bestowing a guarantee for enhanced learning outcomes (Zhoc et al., 2018). There may be other reasons for an insignificant relationship, like if the personnel are expected to solve ambiguous and imprecise problems and then fail to perform, despite the provided autonomy, and fail to learn anything (Kapur, 2008; Sawyer, 2018b). Likewise, the personnel cannot endure creativity and learning whenever they are required to perform far beyond their capabilities (Sawyer, 2018a). Similarly, creativity, provisional failure and learning are interdependent, therefore one can expect transitory failure during the phase of creative performance and learning outcomes (Sawyer, 2011). Furthermore, the learning process is agonizing with a lot of negativity and obstruction (Greene et al., 2019), urging the personnel to abandon the learning phase and creativity altogether (Greene et al., 2012). Moreover, HEIs personnel motivated for creativity and learning pull back in the face of pessimism, disorder, and fear (Zhoc et al., 2018; Greene et al., 2019). This systematic procedure, therefore, asks the HEIs’ teachers to cope with demanding situations with resolve, EI, and learning (Asrar-ul-Haq et al., 2017) and to foster innovation and creative performance (Iqbal et al., 2019). Furthermore, creative performance is used as a mediator (Royston and Reiter-Palmon, 2017) and argues that professionals to hold the continuous EI opportunities (Darvishmotevali et al., 2018), hence, improving their learning outcomes for the ever-changing circumstances of HEIs (Rogaten and Moneta, 2016).

Finally, the considered study investigated the relationship between learning outcomes and OP regarding teachers in Pakistani HEIs. The findings substantiated the argued relationship with an endorsement of Park and Jacobs (2011), however, the mentioned study was conducted in the commercial sector. Furthermore, learning outcomes entails multiple facets i.e., social, cognitive, self-growth outcomes, and satisfaction with university experience, which further argue for multiple subdimensions (Zhoc et al., 2018), necessitating the vibrant efforts for implementing and improving OP, especially of HEIs (Iqbal et al., 2019).

## Conclusion

This study adds value to the EI and learning outcomes literature of HEIs’ teachers by investigating and explaining the teachers’ competence (personal assessment) and creative performance, furthermore, organizational/university performance was also comprehended in relation to learning outcomes. The findings validated that EI, social, cognitive, self-growth outcomes, and satisfaction with the university experience of teachers are valuable for HEIs. Therefore, it is the need of the time that policymakers come up with viable courses of action to successfully implement EI, learning outcomes’ passage, organizational/university performance, competence, and creativity of professionals at HEIs (Zhoc et al., 2018; Iqbal et al., 2019). Furthermore, a clear practical hierarchy within HEIs can convince the individuals to apply and carry forward the implemented policies, hence, fostering the EI, learning outcomes and organizational/university performance substantially. Moreover, HEIs spread recognition, openness, trust, communication and knowledge dissemination (Yasir et al., 2017) which consequently enhance the friendly collegial atmosphere with improved EI, learning outcomes, competence, creativity and organizational/university performance.

### Contributions and Practical Implications

Results of considered research strengthen the theoretical and practical understanding of EI and advocate how HEIs’ teachers can value their competence and creative performance, which in turn enhance their learning outcomes, hence, urging teachers to strive hard for the betterment of overall organizational performance. The current study adds some theoretical and practical implications which are as under;

The findings of this study may avert the consideration of HEIs’ advocators to ponder and strategize incessant roadmaps for the teachers conferring the impulsive educational weights. These findings, furthermore, demand the HEIs align the professional activities in relation to teachers’ EI, their competence (personal assessment), creative performance, and learning outcomes to remodel their spontaneous reactions in impenetrable circumstances for the furtherance of institutions. Moreover, these findings may force the policymakers at HEIs to reconsider, reformat and formulate fresh policies for practice to endure challenges calmly with a well-trained bunch of professionals.

Teachers in HEIs, being a vital part of institutions, should be offered openings to shine in contemporary stimulations (Asrar-ul-Haq et al., 2017). However, there is an apparent downturn in the form of narrow opportunities for training and trained EI personnel, especially teachers, in HEIs (Asrar-ul-Haq et al., 2017), hence, asking for an extensive oration and training of EI for HEIs’ professionals, ultimately bequeaths pledge for the better learning outcomes (Zhoc et al., 2018). This orderly route, moreover, assists the HEIs’ teachers to manage the challenging circumstances with tenacity, EI, and better learning, hence, inducing institutions to perform better (Alam and Ahmad, 2018). Furthermore, teachers’ learning outcomes are vital for the successful implementation and application phase regarding the scheduled plans to nurture university performance and this study tried to contribute to it practically.

### Limitations and Future Research Directions

The considered research is prone to certain limits that ask for an extended investigation. Small size convenience sample from restricted HEIs invites the sample bias and results’ generalizability concerns to other research HEIs. Therefore, future studies should consider a larger sample size with random sampling in order to facilitate the results’ generalizability and ascertain the improved responses to research questions of considered research. Furthermore, a comparative study may also be initiated concerning the public and private HEIs across Pakistan with the explained variables. Second, it is encouraged that future researchers replicate this research in other regions like developing nations to authenticate the extracted results of this research. Third, this study is conducted with a vital focus on academicians; hence, researchers are encouraged to conduct this very study with a main focus on students, thus, replacing the mediators like students’ trust in teachers and learning orientation (commitment to learning) and replacing organizational/university performance with student academic efficacy, graduate employability or student achievement/success/satisfaction.

## Data Availability Statement

The raw data supporting the conclusions of this article will be made available by the authors, without undue reservation.

## Ethics Statement

The studies involving human participants were reviewed and approved by the Northwestern Polytechnical University Research Ethics Review Committee. The participants provided their written informed consent to participate in this study.

## Author Contributions

All authors have substantially contributed and approved for publication.

## Conflict of Interest

The authors declare that the research was conducted in the absence of any commercial or financial relationships that could be construed as a potential conflict of interest.

## Publisher’s Note

All claims expressed in this article are solely those of the authors and do not necessarily represent those of their affiliated organizations, or those of the publisher, the editors and the reviewers. Any product that may be evaluated in this article, or claim that may be made by its manufacturer, is not guaranteed or endorsed by the publisher.
